# Supplementary material for: A fault-intrusion-tolerant system and deadline-aware algorithm for scheduling scientific workflow in the cloud
Source: PeerJ Comput Sci. 2021 Nov 2;7:e747. doi: 10.7717/peerj-cs.747 (PMC8576554; doi:10.7717/peerj-cs.747)
Supplement: Supplemental Information 6 [file peerj-cs-07-747-s006.zip › Myproject1.1/docs/org/cloudbus/cloudsim/provisioners/RamProvisioner.html]

RamProvisioner


JavaScript is disabled on your browser.


- Overview
- Package
- Class
- Use
- Tree
- Deprecated
- Index
- Help

- Prev Class
- Next Class

- Frames
- No Frames

- All Classes

- Summary:
- Nested |
- Field |
- Constr |
- Method

- Detail:
- Field |
- Constr |
- Method


org.cloudbus.cloudsim.provisioners

## Class RamProvisioner

- java.lang.Object
- - org.cloudbus.cloudsim.provisioners.RamProvisioner

- Direct Known Subclasses:
  :   RamProvisionerSimple

  ---

    

  ```
  public abstract class RamProvisioner
  extends java.lang.Object
  ```

  RamProvisioner is an abstract class that represents the provisioning policy of memory to virtual
  machines inside a Host. When extending this class, care must be taken to guarantee that the field
  availableMemory will always contain the amount of free memory available for future allocations.

  Since:
  :   CloudSim Toolkit 1.0

- - ### Constructor Summary

    Constructors

    Constructor and Description

    `RamProvisioner(int ram)`

    Creates the new RamProvisioner.
  - ### Method Summary

    Methods

    | Modifier and Type | Method and Description |
    | --- | --- |
    | `abstract boolean` | `allocateRamForVm(Vm vm, int ram)` Allocates RAM for a given VM. |
    | `void` | `deallocateRamForAllVms()` Releases BW used by a all VMs. |
    | `abstract void` | `deallocateRamForVm(Vm vm)` Releases BW used by a VM. |
    | `abstract int` | `getAllocatedRamForVm(Vm vm)` Gets the allocated RAM for VM. |
    | `int` | `getAvailableRam()` Gets the available RAM in the host. |
    | `int` | `getRam()` Gets the ram. |
    | `int` | `getUsedRam()` Gets the amount of used RAM in the host. |
    | `abstract boolean` | `isSuitableForVm(Vm vm, int ram)` Checks if is suitable for vm. |
    | `protected void` | `setAvailableRam(int availableRam)` Sets the available ram. |
    | `protected void` | `setRam(int ram)` Sets the ram. |

    - ### Methods inherited from class java.lang.Object

      `clone, equals, finalize, getClass, hashCode, notify, notifyAll, toString, wait, wait, wait`

- - ### Constructor Detail


    - #### RamProvisioner

      ```
      public RamProvisioner(int ram)
      ```

      Creates the new RamProvisioner.

      Parameters:
      :   `ram` - the ram
  - ### Method Detail


    - #### allocateRamForVm

      ```
      public abstract boolean allocateRamForVm(Vm vm,
                             int ram)
      ```

      Allocates RAM for a given VM.

      Parameters:
      :   `vm` - virtual machine for which the RAM are being allocated
      :   `ram` - the RAM

      Returns:
      :   $true if the RAM could be allocated; $false otherwise


    - #### getAllocatedRamForVm

      ```
      public abstract int getAllocatedRamForVm(Vm vm)
      ```

      Gets the allocated RAM for VM.

      Parameters:
      :   `vm` - the VM

      Returns:
      :   the allocated RAM for vm


    - #### deallocateRamForVm

      ```
      public abstract void deallocateRamForVm(Vm vm)
      ```

      Releases BW used by a VM.

      Parameters:
      :   `vm` - the vm


    - #### deallocateRamForAllVms

      ```
      public void deallocateRamForAllVms()
      ```

      Releases BW used by a all VMs.


    - #### isSuitableForVm

      ```
      public abstract boolean isSuitableForVm(Vm vm,
                            int ram)
      ```

      Checks if is suitable for vm.

      Parameters:
      :   `vm` - the vm
      :   `ram` - the ram

      Returns:
      :   true, if is suitable for vm


    - #### getRam

      ```
      public int getRam()
      ```

      Gets the ram.

      Returns:
      :   the ram


    - #### setRam

      ```
      protected void setRam(int ram)
      ```

      Sets the ram.

      Parameters:
      :   `ram` - the ram to set


    - #### getUsedRam

      ```
      public int getUsedRam()
      ```

      Gets the amount of used RAM in the host.

      Returns:
      :   used ram


    - #### getAvailableRam

      ```
      public int getAvailableRam()
      ```

      Gets the available RAM in the host.

      Returns:
      :   available ram


    - #### setAvailableRam

      ```
      protected void setAvailableRam(int availableRam)
      ```

      Sets the available ram.

      Parameters:
      :   `availableRam` - the availableRam to set


- Overview
- Package
- Class
- Use
- Tree
- Deprecated
- Index
- Help

- Prev Class
- Next Class

- Frames
- No Frames

- All Classes

- Summary:
- Nested |
- Field |
- Constr |
- Method

- Detail:
- Field |
- Constr |
- Method
